# Virtual Development of Process Parameters for Bulk Metallic Glass Formation in Laser-Based Powder Bed Fusion

**DOI:** 10.3390/ma15020450

**Published:** 2022-01-07

**Authors:** Johan Lindwall, Andreas Lundbäck, Jithin James Marattukalam, Anders Ericsson

**Affiliations:** 1 Department of Engineering Sciences and Mathematics, Solid Mechanics, Luleå University of Technology, 97187 Lulea, Sweden; andreas.lundback@ltu.se; 2 Department of Physics, Materials Physics, Uppsala University, P.O. Box 530, 75121 Uppsala, Sweden; jithin.marattukalam@physics.uu.se; 3 Division of Solid Mechanics, Lund University, P.O. Box 118, 22100 Lund, Sweden; anders.ericsson@solid.lth.se

**Keywords:** additive manufacturing, simulation of laser-based powder bed fusion, metallic glass, crystallisation in metallic glass, classical nucleation and growth theory

## Abstract

The development of process parameters and scanning strategies for bulk metallic glass formation during additive manufacturing is time-consuming and costly. It typically involves trials with varying settings and destructive testing to evaluate the final phase structure of the experimental samples. In this study, we present an alternative method by modelling to predict the influence of the process parameters on the crystalline phase evolution during laser-based powder bed fusion (PBF-LB). The methodology is demonstrated by performing simulations, varying the following parameters: laser power, hatch spacing and hatch length. The results are compared in terms of crystalline volume fraction, crystal number density and mean crystal radius after scanning five consecutive layers. The result from the simulation shows an identical trend for the predicted crystalline phase fraction compared to the experimental estimates. It is shown that a low laser power, large hatch spacing and long hatch lengths are beneficial for glass formation during PBF-LB. The absolute values show an offset though, over-predicted by the numerical model. The method can indicate favourable parameter settings and be a complementary tool in the development of scanning strategies and processing parameters for additive manufacturing of bulk metallic glass.

## 1. Introduction

This study presents a method to assist the development of process parameters by simulation in laser-based powder bed fusion (PBF-LB) to benefit bulk metallic glass formation. The method includes transient modelling of the temperature field arising from the laser scanning using a finite element (FE) model and phase transformation modelling of crystalline precipitate formation in the metallic glass using the classical nucleation and growth theory (CNT). Simulation of different process parameters with this method enables the prediction of favourable parameters for bulk metallic glass formation. The method can be used to reduce the number of trials in the time-consuming trial and error process of finding process parameters. It can also be a powerful tool to assist the fine-tuning of process parameters and scanning strategies in PBF-LB.

Metallic glasses have an amorphous atomic structure that results in very different mechanical properties as compared to their crystalline counterparts and other high-performance steels [1,2,3]. Metallic glasses generally possess very high elastic strain and yield stress limit, very good corrosion and wear resistance, but poor fracture toughness [4]. Metallic glass is generally very brittle and deforms plastically by localised strain in the form of shear bend. Control of shear band initiation and propagation has been demonstrated to improve the plasticity and fatigue behaviour of metallic glass [5,6]. Di et al. [7] further showed that the plasticity could be enhanced by making an amorphous-crystalline composite in which the crystalline particles may inhibit the propagation of shear bands, resulting in increased ductility. Metallic glass can be produced by rapid solidification of the molten metal to bypass crystallisation. The required cooling rate to bypass crystallisation can be up to $10^{10}\;{\mathrm{Ks}}^{-1}$, depending on the material [8]. By alloy design, the critical cooling rate can be reduced to about $10^{-1}$–$10^{3}\;{\mathrm{Ks}}^{-1}$ for alloys with good glass-forming ability [2]. For the present material, the conventional alloy AMZ4 Zr${}_{59.3}$Cu${}_{28.8}$Al${}_{10.4}$Nb${}_{1.5}$ (at%) is an alloy designed for metallic glass formation, and the critical cooling rate is estimated to 2500 K/s [9]. Because of the necessity of rapid cooling, traditional casting techniques are limited to relatively small volumes. Melt spinning is a technique for producing metallic glass, but it is geometrically restricted to thin ribbons [1]. Additive manufacturing (AM) with PBF-LB on the other hand can be used to overcome the size and shape limitations by continuous rapid solidification of a very small melt pool [10]. However, there is a potential risk for crystallisation in the heat affected zone (HAZ) in the material produced by PBF-LB—mainly because of reheating from adjacent laser scans and subsequent layers [11,12].

Development of process parameters and scanning strategies to reduce the evolution of crystalline precipitates during PBF-LB can be a tedious and costly process [12]. The traditional process involves testing of various parameters and evaluation of a small volume by preparation of samples and analysis using e.g., scanning electron microscopy (SEM) or X-ray diffraction (XRD) [13,14,15]. The experimental analysis is typically performed after processing and may not entirely explain the formation of crystalline phases nor the temperature history that enables the phase transformation. A complementary method for the prediction of crystallisation during the PBF-LB process is by modelling the temperature field and phase transformation [16,17]. Shen et al. [18] developed a simplified model based on classical nucleation and growth theory to calculate the evolution of a crystalline structure with steady-state nucleation during laser heat treatment. The model enables the prediction of the crystalline volume fraction and size of the HAZ after one laser pass. Another model, based on CNT, was developed by Ericsson et al. [19] and examines the effect of transient nucleation during the temperature history by PBF-LB. The CNT models are computationally efficient and can be coupled to FE-analysis of the temperature field. This method can be a useful tool that can help to understand the evolution of crystalline precipitates during PBF-LB.

Simulation of the transient thermal field in the HAZ by FE-modelling requires a fine mesh grid in close proximity of the fusion zone and high temporal resolution. In addition, a calibrated heat input model and thermodynamic material data are also required for reliable predictions. Modelling of the scanning strategy over the course of many layers further increases the complexity because of the large differences in temporal and spatial scales. These challenges are addressed by sub-modelling with local mesh adaptivity on a confined domain. The transient behaviour of the applied energy is adopted during the simulation to achieve a good representation of the temperature history in the HAZ. Current studies are generally limited to simulations of one laser string, remelted multiple times. The current model enables the simulation of many hatches and multiple layers and can calculate the effect of different process parameters and scanning strategies.

The method presented in this study includes a CNT model coupled to a thermal FE-model to predict the evolution of crystalline precipitates during the PBF-LB process. Simulations are performed with varying process parameters to demonstrate the potential as a tool for the virtual development of parameters for glass formation. The study includes a variation of three parameters: laser power, hatch spacing and hatch length. The hatch spacing is the distance between two adjacent laser tracks and the hatch length is the travelling distance of the laser spot in each track. The values chosen in this study for demonstration purposes are 80 and 100 μm for the hatch spacing and 4 to 6 mm for the hatch length. The work includes computation of the crystalline volume fraction by the effect of the laser power and the simulation results are compared to experimental estimates from Marattukalam et al. [15]. The laser power is varied between 75 and 105 W. The simulations are performed with scanning of five layers including ten adjacent hatches and a re-heating scanning strategy, consistent with the development of process parameters in [15].

## 2. Modelling of Phase Evolution in PBF-LB

Simulation of the phase evolution during the PBF-LB process is performed with a classical nucleation and growth theory model coupled to a thermal finite element model based on Lindwall et al. [20]. The crystalline phase evolution is computed in a staggered approach directly in the FE software after each converged thermal increment. The thermal model is developed in MSC Marc with user-defined subroutines to model the heat source, the scanning strategy as well as the crystalline phase evolution during the PBF-LB process. The modelling domain in current study is limited to a 6 × 6 × 2 mm volume (build area and thickness, respectively) and includes the complete scanning paths of a limited number of hatches and five layers. The domain is discretized with three-dimensional, eight-noded solid elements. All elements that belong to a layer are inactivated at the start of the simulation. The elements are activated on a layer-by-layer basis during the re-coating cycle between the layers. The element side lengths are 80 μm in the bulk of the domain. A refined mesh grid is used close to the top surface to resolve the layer thickness, which is 20 μm. Elements close to the centre of the plane in the build direction are refined by local mesh adaptivity that reduces the element side lengths to 5 μm in all directions. This refined region is the modelling domain for the phase transformation model and consists of about 131,000 elements and 1,049,000 integration points. It is also used as a control volume in which the evolution of crystalline precipitates is computed.

**Figure 1:**
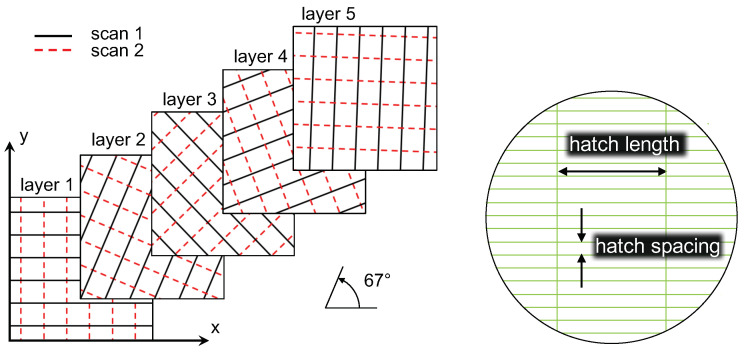
(**left**) Illustration of scanning strategy with dual scanning within each layer and an angular rotation of 67° between the layers. The solid and dashed lines represent paths of the first and second scan sequence, respectively; (**right**) Illustration of the hatch length and hatch spacing.

A cooling time of 10 s is included in the simulation between the activation of each new layer. The cooling time represents the processing time of the surrounding material and the process where a new layer of powder is spread. During this time, the heat of the molten layer dissipates, allowing it to cool down prior to the subsequent heating sequence. Because of the reduced modelling domain, where the substrate is excluded, a boundary condition with a fixed temperature of 28 °C is applied at the bottom surface during the cooling time between the layers [21]. This ensures that the thermal energy is not constantly increasing in the material, which can cause an unrealistic accumulation of heat. The boundary condition is a sufficient approximation provided that the thickness of the modelling domain is large enough to allow heat dissipation to room temperature. All simulations are performed with the scanning strategy and optimal process parameters developed by Marattukalam et al. [15]. The layer thickness is 20 μm and the scanning velocity is 2000 mm/s. The scanning strategy includes re-melting of each layer, where the layer is scanned twice with a rotation of 90° between the scanning vectors. The scanning vectors are rotated 67° between each layer as illustrated in Figure 1 (left). The approach to scan a layer is illustrated in Figure 1 (right).

### 2.1. Thermal Model

Thermal modelling includes a heat source that distributes the heat produced by the laser source. The heat input from the laser heat source is focused on a spot size of 40 μm [15], but the interaction with powder/melt pool and the effect of convective flow distributes the energy to form a larger melt pool size. In the thermal model, the effect of convective flow is modelled by applying a heat source of similar size to the melt pool. As a result of this approximation, the heat source model needs to be calibrated to fit the actual heat distribution.

The heat source is further simplified by temporal reduction to enable simulation of the temperature field by multiple laser tracks and several layers [22]. The total heat in a laser track that passes outside of the control volume is added simultaneously in a string-by-string approach. This reduces the temperature history of the laser scan to only one increment and reduces the computational time. Conservation of energy ensures that the total thermal energy is not modified by the temporal reduction. The heat input of laser tracks that pass through the control volume is added by sweeping the heat source along the scanning vectors. The model of the heat input used in this study is based on the commonly used three-dimensional Gaussian heat source by Goldak et al. [23], reduced to a uniform distribution in the scanning direction (Equation (1)):

(1) $$q\left(y,z\right)=\frac{3Q\eta }{\pi c{\left(d/2\right)}^{2}}\exp\left(-\frac{3y^{2}}{{\left(d/2\right)}^{2}}-\frac{3z^{2}}{c^{2}}\right)$$

The parameter *Q* is the laser power, varied between 75 and 105 W during the simulations and η is the laser power absorption, chosen at 0.35. The depth and width of the heat source are determined by c=35 μm and d=70 μm, based on observations by SEM-imaging in a cross-section of a single laser scan in Lindwall et al. [20]. The variables yandz are the local coordinates of the transverse and downwards direction, respectively, with origin in the centre of the heat source. The longitudinal dimension of the heat source is either the hatch length (when string-by-string heating applies) or the same as the width (when swept along the laser path).

**Figure 2:**
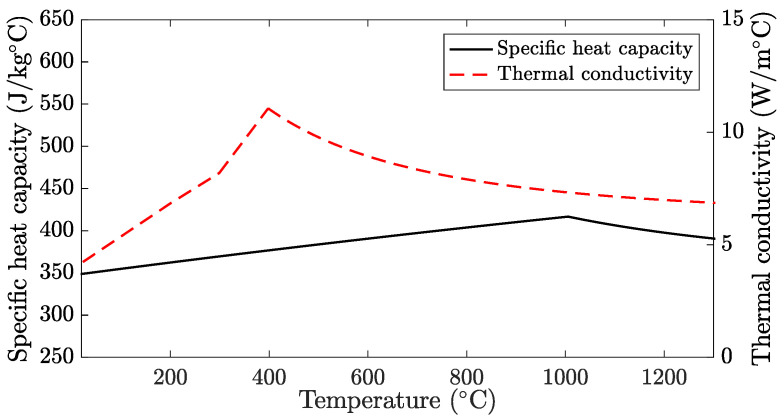
Thermal material data used in the simulations based on Lindwall et al. [17], Heinrich et al. [24] and Lindwall et al. [20].

The material data used in the simulations are shown in Figure 2. AMZ4 has a liquidus temperature of 930 °C and begins to crystallise at approximately 500 °C during moderate heating from the amorphous solid phase Lindwall et al. [17]. A model for the specific heat capacity of the amorphous material AMZ4 was developed in previous work Lindwall et al. [17], based on differential scanning calorimetry measurements by Heinrich et al. [24]. The measurements were performed during heating with 0.333 K/s and the data were fitted to analytical expressions for the heat capacity at temperatures above and below the glass transition temperature (400 °C). With the high heating and cooling rates in PBF-LB, the glass transition region will pass very quickly. Therefore, the material model is modified in the present work by ignoring the effect on the heat capacity caused by glass transformation. The specific heat capacity is instead treated as solid glass from room temperature to the liquidus temperature.

The thermal conductivity of the amorphous material was measured in [20] using the transient plane source (HotDisk) method at temperatures ranging from room temperature to 300 °C. At higher temperatures, the thermal conductivity is approximated by $k=\rho c_{p}\alpha$, where ρ is the material density, $c_{p}$ the specific heat capacity based on Lindwall et al. [17] and Heinrich et al. [24], and α are the measured thermal diffusivity at 300 °C, which is assumed to be constant at higher temperatures [25].

### 2.2. Phase Transformation Model

The crystalline phase evolution is computed using a model based on classical nucleation and growth theory, developed by Ericsson et al. [19]. The model computes the formation and growth of crystalline precipitates by numerically solving the evolution of the crystal size distribution. Assuming steady-state nucleation, the model is implemented in the FE-model to compute the size distribution at all integration points in the control volume in a staggered approach. Each integration point has an associated structure component that contains state variables that stores the number density of crystals and the corresponding sizes. The state variables contain all information of the crystal size distribution in a representative volume corresponding to the integration point. After each time step, newly formed nuclei are inserted into the size distribution and the size of the crystals are updated by making use of the growth rate. This approach follows the Lagrange-like numerical scheme described in [26] and is solved in sub increments within the thermal model. For a more detailed description of the model, the reader is referred to Ericsson et al. [19]. Note that the same model parameters are used in the present study.

The CNT model evaluates the change in Gibbs energy ΔG(n), required to form a crystalline cluster of *n* atoms, given by (Equation (2)) [27]

(2) $$\Delta G\left(n\right)=n\Delta G^{\prime }+{\left(36\pi \right)}^{1/3}{\bar{v}}^{2/3}n^{2/3}\sigma$$

where $\Delta G^{\prime }<0$ is the change in Gibbs energy per atom, $\bar{v}$ is the average atomic volume, and σ is the interfacial energy per unit area between the two phases. Equation (2) shows a maximum at the critical cluster size $n^{*}$, above which the crystals are stable and will grow to larger sizes. The number of crystals that forms during an increment is approximated by the steady-state nucleation rate, expressed as (Equation (3)) [26,28,29]

(3) $$I^{st}=N_{0}Zk^{+}\left(n^{*}\right)\exp\left(-\frac{\Delta G(n^{*})}{k_{B}T}\right)$$

where $N_{0}$ is the atomic number density in the system, *Z* is the Zeldovich factor, $k^{+}\left(n^{*}\right)$ is the atomic attachment rate, $\Delta G(n^{*})$ the change in Gibbs energy, evaluated at the critical nuclei size, $n^{*}$, $k_{B}$ is the Boltzmann constant, and *T* the absolute temperature. The number density of crystals that forms during an increment is calculated by integration of the temperature-dependent nucleation rate with respect to time using the trapezoidal rule. The newly formed crystals are stored in the state variables and the radius, *r* of all crystals is updated by calculating the growth using an explicit time-stepping scheme (Equation (4)) [26,28]

(4) $$r\left(t_{i}\right)=r\left(t_{i-1}\right)+u\left(r\left(t_{i-1}\right)\right)\Delta t$$

where Δt is the time step of the sub-increment and u(r) is the growth rate. A polymorphic growth is assumed and the growth rate is expressed as (Equation (5)) [28]

(5) $$u\left(r\right)=\frac{16D}{\lambda^{2}}{\left(\frac{3\bar{v}}{4\pi }\right)}^{1/3}\sinh\left[\frac{\bar{v}}{2k_{B}T}\left(|\Delta G_{v}|-\frac{2\sigma }{r}\right)\right]$$

where *D* is the diffusivity, λ is the atomic jump distance and $\Delta G_{v}$ describes the change in volumetric Gibbs energy between the crystalline and undercooled liquid state. The crystalline volume fraction is computed using the Johnson–Mehl–Avrami–Kolmogorov (JMAK) equation [30,31,32] expressed as (Equation (6))

(6) $$x\left(t\right)=1-\exp\left(-\frac{4\pi }{3}\sum_{i=1}^{m}\hat{N_{i}}r_{i}^{3}\right)$$

where ${\hat{N}}_{i}$ is the number density of crystals formed during time increment *i* and $r_{i}$ is the radius of the corresponding crystals. As previously described, these quantities are available from the state variables stored throughout the simulation.

## 3. Demonstration Methodology

The model is used to simulate the resulting crystallisation for different combinations of process parameters. The computed crystalline volume fraction is compared with experimental estimates obtained as part of the process development of alloy AMZ4 by Marattukalam et al. [15]. In the experimental study by Marattukalam et al. [15], a total of 64 samples were fabricated using an EOS M100 (EOS GmbH, Krailling, Germany) with a wide variation of laser process parameters. The samples were investigated by X-ray diffraction (XRD) using a Cu-Kα radiation, Bruker (Bruker AXS GmbH, Karlsruhe, Germany) D8 advanced diffractometer to determine the presence of crystals. The crystalline volume fraction was estimated from the XRD analysis by calculating the ratio of the area of the crystalline peaks and the amorphous phase using TOPAS software (version 6) for Rietveld refinement (Bruker). For further details of the sample production and characterisation, the reader is referred to Marattukalam et al. [15].

The laser power has been shown to have a direct effect on the crystalline volume fraction [15]. With a laser power of 80 W, a small amount of crystalline volume fraction was detected by XRD. Increased laser power resulted in increased crystalline volume fraction, and reduced laser power resulted in no visible crystalline structure. The effect of the laser power is therefore simulated and compared with the experimental estimates. The laser power is increased in steps of 5 W from 75 to 105 W, while the hatch spacing and hatch length are kept constant at 100 μm and 5 mm, respectively.

**Figure 3:**
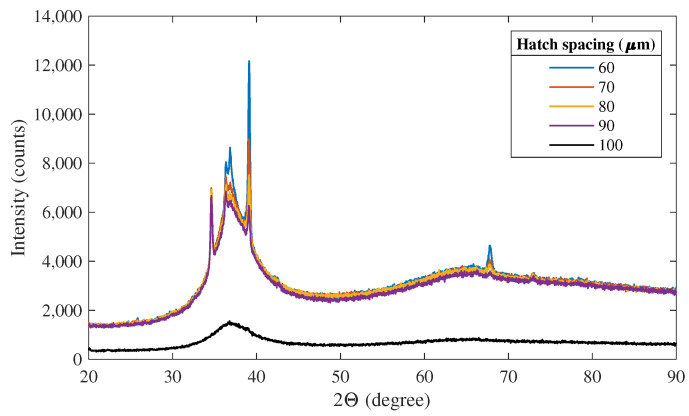
XRD scattering pattern of printed samples with varying hatch spacing [15]. The laser power and hatch length were kept constant at 80 W and 5 mm, respectively.

The second parameter that is varied in the simulations is the hatch spacing, which defines the spacing between adjacent scan paths. This parameter is varied between 60 and 100 μm in steps on 10 μm. A smaller hatch spacing provides more energy to the material than a larger hatch spacing as a larger number of laser scans are needed to complete the layer. This results in a higher crystalline volume fraction as seen in the results of XRD-analysis from [15] shown in Figure 3.

The third parameter that is examined is the hatch length, which defines the total length of the scanning vectors. The hatch length is varied between 4 and 6 mm in steps of one millimetre, for different hatch spacings of 80 and 100 μm and a constant laser power of 80 W. A shorter hatch length reduces the time before reheating from an adjacent laser scan. This is expected to result in an increased peak temperature by the reheating because of the reduced time for heat dissipation. The evolution of crystalline precipitates is calculated during the simulation, and the influence of the parameters on crystallisation is examined.

**Figure 4:**
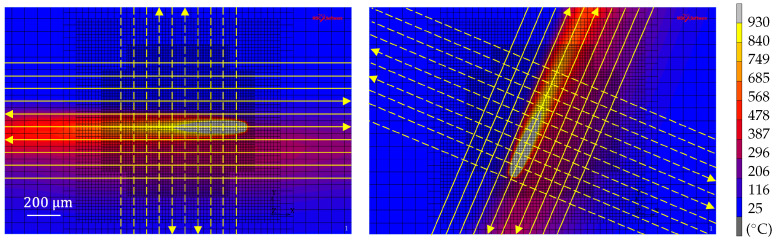
Illustration of the scanning strategy in layers one and two showing the laser scanning vectors for the first and second scan (solid and dashed lines, respectively). The scanning strategy is rotated 67° between the layers. The colour contours show the simulated temperature field (°C) from room temperature to the melting temperature of 930 °C.

In the simulation, the total heat input in each layer is reduced to ten adjacent hatches evenly distributed around the centre point of the control volume, as illustrated in Figure 4. The figure shows the scanning vectors of the first scan in layers one and two (solid lines), and the re-heating from the second scan (dashed lines). The arrows indicate the direction of paths with a transient heat source, whereas lines (without arrowhead) are heated instantly along the whole scan line.

The phase transformation model by CNT is applied to all integration points in the control volume during the simulation. In each integration point, the number density of crystals, the mean crystal radius and the crystalline volume fraction are calculated and stored throughout the simulation.

## 4. Simulation Results

The crystalline phase evolution is predicted for the different sets of process parameters. The phase transformation model computes the crystalline volume fraction, the mean crystal radius and the number density of crystals at all 1,049,000 integration points in the control volume. The result in the integration points is extrapolated to the nearest node in the element where nodal averaging is applied. The presented results are the mean value on all nodes in the control volume.

### 4.1. Laser Power

**Figure 5:**
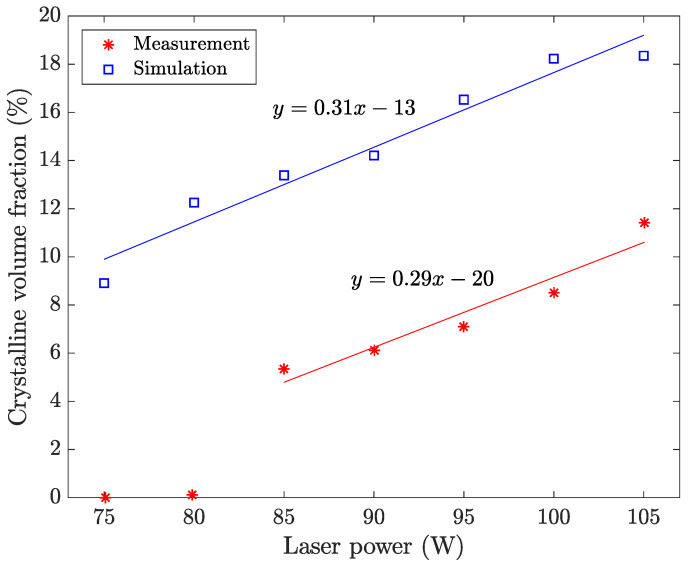
Simulated and measured crystalline volume fraction [15] with respect to laser power. Hatch spacing and hatch length are constant at 100 μm and 5 mm, respectively. The R^2^ value for the fitted lines are 0.92 and 0.96 for the measurement and simulation, respectively. The experimental values for the 75 and 80 W samples were omitted from the fitting procedure as local fractions of crystals were apparent from microscopy analysis [15].

The calculated mean crystalline volume fraction by the change of laser power is presented in Figure 5. The calculated crystalline volume fractions are compared to the estimated values by Marattukalam et al. [15]. Here, the hatch spacing and hatch length were kept constant at 100 μm and 5 mm, respectively. The simulated and experimental results were fitted to linear functions to obtain trend lines.

### 4.2. Hatch Spacing

**Figure 6:**
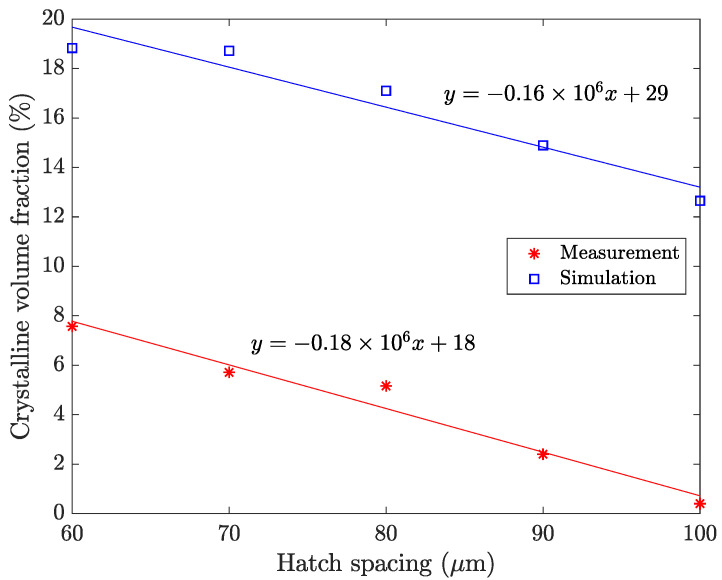
Simulated and experimentally estimated crystalline volume fraction with respect to hatch spacing. Laser power and hatch length are constant at 80 W and 5 mm, respectively. The R^2^ value for the fitted lines are 0.97 and 0.93 for the measurement and simulation, respectively.

The resulting crystalline volume fraction is shown in Figure 6 together with the estimated crystalline volume fraction from the XRD analysis. The hatch spacing is varied between 60 to 100 μm in the steps of 10 μm. The laser power is 80 W and the hatch length is 5 mm. The simulations indicate a decrease in crystalline volume fraction with an increased hatch spacing. The same trend is observed in the experimental data.

### 4.3. Hatch Length and Hatch Spacing

**Figure 7:**
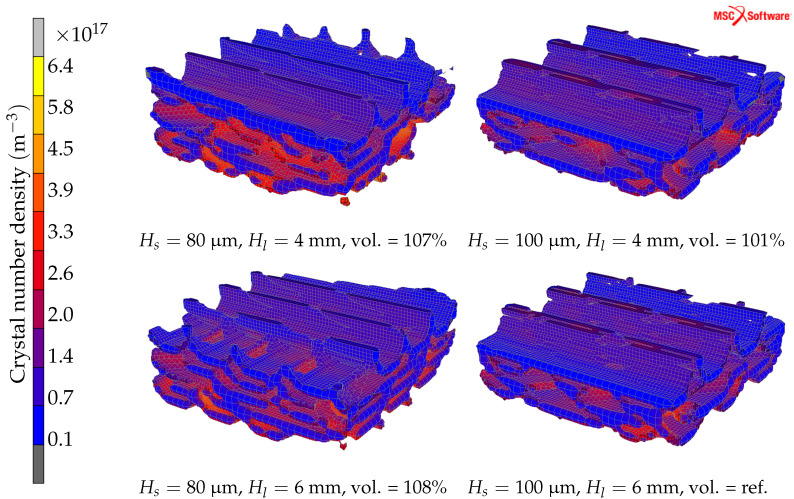
Finite elements in the control volume with a crystalline volume fraction ≥10%. Colour contour shows the crystal number density (m^−3^), $H_{s}$— hatch spacing, $H_{l}$—hatch length, vol. are the relative size of this quantity with respect to the reference with the least element volume.

The effect of hatch length using different hatch spacings is evaluated using a constant laser power of 80 W. The hatch length and hatch spacing are varied between 4–6 mm and 80–100 μm, respectively. All finite elements with a final crystalline volume fraction ≥10% by four different process parameters are shown in Figure 7. The smallest volume (fewest elements) is predicted with a hatch spacing of 100 μm and a hatch length of 6 mm. A hatch spacing of 80 μm results in a 7–8% larger volume. The change in hatch length results in a change in the volume by 1%. The colour contour in Figure 7 shows the computed crystal number density in the control volume.

The mean crystalline volume fraction is computed at all 1,049,000 data points in the control volume, and the results are presented in Table 1. The lowest crystalline volume fraction is obtained with the largest hatch spacing and hatch length. The mean crystalline volume fraction in the reference setting is calculated to 12.6%. This value is reduced to 11.8% when the hatch length is increased by one millimetre. The highest crystalline volume fraction is obtained with a hatch spacing of 80 μm and no clear dependence on the hatch length is observed. The mean crystalline volume fraction is about 17% and 1.35 times larger than the reference value with a hatch spacing of 80 μm and hatch length 5 mm.

The calculated mean number density of crystals in the control volume is presented in Table 2. The results follow the same trend as the crystalline volume fraction upon changes in hatch spacing but also show a dependence on the hatch length. More crystals form with a narrow hatch spacing and hatch length. The highest value is predicted with a hatch spacing of 80 μm and a hatch length of 4 mm, which resulted in 1.66 times more crystals relative to the reference simulation using the settings in [15], i.e., 1.81 $\times \;10^{17}\;m^{-3}$. By increasing the hatch length to 6 mm, the mean number density is reduced to 1.66 $\times \;10^{17}\;m^{-3}$.

The resulting mean radius of the crystals is presented in Table 3. The trend is consistent with previous results and the smallest mean crystal radius is achieved with the widest hatch spacing and hatch length. The largest mean radius is computed to 179 nm, with a hatch spacing of 80 μm and hatch length of 4 mm.

## 5. Discussion

The crystalline volume fraction increases with increased laser power. With a higher laser power, more energy is absorbed by the material, making the melt pool larger with increased peak temperature. This leads to lower cooling rates and increased size of the HAZ, allowing for more time for nucleation and growth of crystals. The result from the simulations predicts the same trend as for the manufactured samples in Marattukalam et al. [15]. The crystalline volume fraction is higher in the simulations. One possible reason is that the assumption of steady-state nucleation overestimates the nucleation rate for the high heating and cooling rates that occurs in the AM process [19]. Furthermore, the nucleation and growth of crystals are assumed to occur under polymorphic conditions and the influence of chemical redistribution during nucleation and growth are not considered. Both the assumption of steady-state nucleation and polymorphic conditions result in a higher rate of crystallisation and a difference between the simulated and measured results is therefore expected. The sensitivity of model parameters such as e.g., the interfacial energy, which shows a strong dependence on the nucleation rate, will of course also influence the simulated crystallisation. Another uncertainty is the resolution of the XRD equipment. Laboratory XRD equipment has been reported by Sohrabi et al. [33] to not be able to detect nanocrystals in the HAZ, which could explain the threshold shown in Figure 5. Nevertheless, the simulated results show a satisfactory agreement with the experimental data, which shows that the model can capture the trend in crystallisation when the process parameters are changed.

The change in crystalline volume fraction due to variation of hatch spacing is also consistent between the simulated result and experimental estimates. A narrower hatch spacing results in a higher crystalline volume fraction. This is a direct consequence of the increased total energy input, which leads to higher temperatures and increased size of the HAZ. Increased temperatures will benefit the growth of crystals formed from previous thermal cycles which results in a higher crystalline volume fraction. The reduced hatch spacing will also cause more overlap between the HAZ from adjacent hatches, resulting in more pronounced reheating of already solidified material. The overlapping regions result in increased crystallisation as more crystals form and grow for each thermal cycle.

The influence of hatch length is examined using two different values of the hatch spacing. The resulting mean crystal number density and mean crystal radius are consistent and both values increase with a reduced hatch length. With a reduced hatch length, the time between the scanning of two adjacent tracks is shorter. This affects the temperature history in the HAZ because of the reduced time for heat dissipation, resulting in increased crystallisation.

The crystalline volume fraction shows a clear dependency on the hatch length using a 100 μm hatch spacing but not for the 80 μm hatch spacing. With a hatch spacing of 100 μm and changing the hatch length from 5 to 6 mm, the crystalline volume fraction was reduced by 7%. With a hatch spacing of 80 μm, the computed crystalline volume fraction is not affected by the variation of hatch length. The results suggest that the influence of the hatch length on crystallisation is less pronounced than the hatch spacing. The result is reasonable considering that a reduced hatch spacing results in more overlap of the HAZ between the laser scans, leading to higher temperatures during reheating. This effect shows a more pronounced influence on crystallisation compared to the reduced time for heat dissipation arising from a shorter hatch length.

The computation time (CPU time) for one simulation with ten adjacent laser hatches and dual scanning on five layers is about one week and includes about 3000 time steps. The simulations were performed with multi-threading on eight cores for assembly and matrix solving, which reduced the simulation time to just above two days. Thus, the time for the calibration of process parameters can be significantly reduced as many simulations can be performed simultaneously in a relatively short time.

The results demonstrate that the presented modelling approach can aid the development of process parameters in the early stages for rough estimations of the impact of the parameters on crystallisation. The simulations may also be useful during fine-tuning of process parameters and scanning strategies to support the glass formation during PBF-LB. The simulations can also help to understand where and when crystals are formed and identify the thermal conditions that result in crystallisation.

## 6. Conclusions

In this study, a modelling approach for simulation of the crystalline phase evolution in a metallic glass during PBF-LB is presented. The evolution of cluster number density, cluster size and crystalline volume fraction is predicted by the influence of process parameters. The parameters that are evaluated are the laser power, hatch spacing and hatch length. The temperature field that drives the crystallisation is computed by finite element analysis on a three-dimensional domain including five layers and a representative number of hatches. The scanning strategy includes reheating in each layer by a 90° rotation of the laser scanning vectors, and a 67° rotation of the scanning vectors between each layer.

In the simulations, the phase transformation is modelled using classical nucleation and growth theory with the assumption of steady-state nucleation. The CNT model calculates the crystalline phase evolution in the three-dimensional domain at 1,049,000 positions with a spatial resolution of about 2.5 μm. The resulting crystalline volume fraction, the number density of crystalline clusters and the radius of the clusters are computed by the mean of all data points.

The simulations enable comparison of the crystalline size distribution as a result of the process parameters and scanning strategies during manufacturing of bulk metallic glass with PBF-LB. The model is shown to accurately predict the trend in crystallisation with respect to laser power and hatch spacing. The results indicated that a low laser power, large hatch spacing and long hatch lengths are beneficial for glass formation during PBF-LB. The results demonstrate that simulations can be a time and cost-saving tool during the development and calibration of process parameters for manufacturing of glass forming materials with low crystallinity.

## Figures and Tables

**Table 1:** Mean crystalline volume fraction in control volume.

	Hatch Spacing	80 μm	100 μm
Hatch Length			
4 mm		17.0%	12.8%
5 mm		17.1%	12.6%
6 mm		17.0%	11.8%

**Table 2:** Mean number density in control volume.

	Hatch Spacing	80 μm	100 μm
Hatch Length			
4 mm		3.01 $\times \;10^{17}\;m^{-3}$	1.85 $\times \;10^{17}\;m^{-3}$
5 mm		2.96 $\times \;10^{17}\;m^{-3}$	1.8 $\times \;10^{17}\;m^{-3}$
6 mm		2.68 $\times \;10^{17}\;m^{-3}$	1.66 $\times \;10^{17}\;m^{-3}$

**Table 3:** Mean crystal radius in control volume.

	Hatch Spacing	80 μm	100 μm
Hatch Length			
4 mm		179 nm	143 nm
5 mm		176 nm	139 nm
6 mm		175 nm	131 nm

## Data Availability

The data presented in this study are available upon request from the corresponding author.
